# Factors determining postoperative and outpatient follow-up period in patients undergoing single lumbar disc herniation

**DOI:** 10.25122/jml-2023-0288

**Published:** 2023-10

**Authors:** Abbas Abdulameer Kadhim, Wissam Saleh Hakim, Ali Saleh Aljanabi

**Affiliations:** 1 Department of Surgery, College of Medicine, University of Al-Qadisiyah, Al Diwaniyah, Qadisiyyah Province, Iraq

**Keywords:** post-operative follow, lumbar disc herniation, discectomy

## Abstract

The precise evaluation of postoperative outcomes in patients with lumbar disc surgery is quite difficult since the pre-operative factors and patient responses differ. Several questionnaires assess the outcome of herniated lumbar disc surgeries. However, the clinical outcome may vary widely, indicating the significance of precise preoperative assessments to ensure better outcome prediction. Previous long-term studies suggest fewer positive outcomes in cases with prolonged preoperative history. In the present retrospective study, we aimed to assess the outcome of patients with lumbar discectomy in Iraqi patients by evaluating the surgical outcome. This research was performed in the orthopedic center of the Teaching Hospital of Adiwaniyah Province, Iraq. The study was based on retrieving hospital records of patients who were subjected to surgical intervention for lumbar disc herniation from 2018 to 2022. The sample consisted of patients with lumbar disc herniation who were subjected to discectomy at one level even in cases where both approaches were used. Age, gender, income, education level, and degree of disc involvement did not significantly correlate with the type of surgical approach (p>0.05); however, there were significant positive correlations to body mass index and duration of disease (p<0.05). Therefore, the body mass index and duration of disease are significant predictors of prolonged postoperative follow-up and hospital stay duration.

## INTRODUCTION

Herniation of intervertebral disc refers to the displacement of the disc material out of its normal position, wherein the prevalence is highest at the lumbar site. It commonly occurs in middle-aged patients and young adults, who are the majority of surgery candidates [1]. The incidence of lumbar disc herniation may be underestimated, as many unreported cases may be asymptomatic or symptoms resolved without intervention [2].

Several factors are attributed to disc herniation; the most common of which is the degenerative disease of the disc [3]. Degeneration of the disc is a multifactorial complex condition in which both genetic and environmental factors (e.g., heavy physical work, trauma, psychosocial stress) play a role [2]. However, it increases with age, and the incidence of herniated lumbar disc is more common in the middle-aged group [3, 4]. In the last few decades, researchers have shown that genetic predisposition may be the prime risk in the etiology of degeneration rather than the physical factors [3].

Nonetheless, surgery is still the option in cases when conservative management has failed, namely when the patient has no significant pain remission over 4-12 weeks, when weakness in motor activity or intestinal or bladder dysfunctions are present [4].

The first reported operation of discectomy of the lumbar disc dates back to the 1920s, making it the most encountered spine surgery in the world [5]. These surgeries present great results, with success rates between 49% and 90% [6, 7]. However, the clinical outcome may vary widely, indicating the importance of precise preoperative assessments to ensure better prediction of outcome, as previous studies suggest less positive outcomes in cases with prolonged preoperative history [8].

The precise evaluation of postoperative outcomes in patients with lumbar disc surgery is quite difficult since the pre-operative factors and patient responses differ. Several questionnaires assessed the outcome of herniated lumbar disc surgeries. The Oracle Data Integrator (ODI) system was regarded as having a significant correlation between outcome and surgical procedure [9-10]. The patient’s occupation influences surgery outcome and, hence, the long-lasting post-operative symptoms. Patients working in physically demanding fields face more disability in comparison to other occupations. Heavy physical work leads to an increased prevalence of torsion stress and flexion and extension stresses on the spine, which promote significant increases in the shearing and loading forces applied to the spinal column with an impact the disc degeneration and injuries to the facet joints. However, some studies deny the direct relation between outcome and occupation. This may be attributed to differences in the classifying workloads and assessment of the outcome [11, 12]. Previous research reported several aspects associated with the prediction of good surgical outcomes, especially patient age, as young patients have better outcomes. The duration of the symptoms also plays a role, with a shorter duration being associated the better outcomes [13]. A poor outcome can mean the recurrence of symptoms or inability to re-engage in previous day-to-day activities and may be attributed to facet joint involvement and the loss of intervertebral disc height [14]. This aggravates the pressure on the roots of the nerves. The association between outcome and gender remains controversial, as some studies showed worse outcomes in women compared to men [15]. In the present study, we aimed to assess the outcomes of lumbar discectomy in Iraqi patients.

## MATERIAL AND METHODS

### Data collection

The present retrospective study was performed in the orthopedic center in the Teaching Hospital of Adiwaniyah Province, Iraq. Hospital records from 2018 to 2022 were retrieved and analyzed. The sample consisted of patients with lumber disc herniation who were subjected to discectomy at a single level even if the approach was bilateral. The exclusion criteria were as follows: spondylolysis and spondylolisthesis, postoperative hematoma or infection, severe neurologic deficit, trauma after discectomy, multilevel discectomy, recurrence of herniation after surgery, dynamic fixation or motion segment preservation, or any other interventions and previous discectomy history.

Patients’ records were searched for the outpatient follow-up period (OFP), postoperative hospitalization period (PHP), and preoperative symptom duration. The follow-up period was restricted to six months. The associations of sociodemographic factors, clinical characteristics, and imaging characteristics to the follow-up period (OFP) were evaluated. These variables included age, gender, body mass index (BMI), educational level, income, surgical approach, the presence of motor weakness, the preoperative conservative treatment period, the severity of root compression, disc degeneration, and the disc level.

### Statistical analysis

Data were analyzed using a statistical package for social sciences (SPSS, Chicago, USA, IBM, version 16.0). Some variables were outlined as numbers and percentages, while others were shown as range, standard deviation, and mean. Data analysis involved a Pearson correlation, using a p≤0.05 level of significance.

## RESULTS

**Figure 1:**
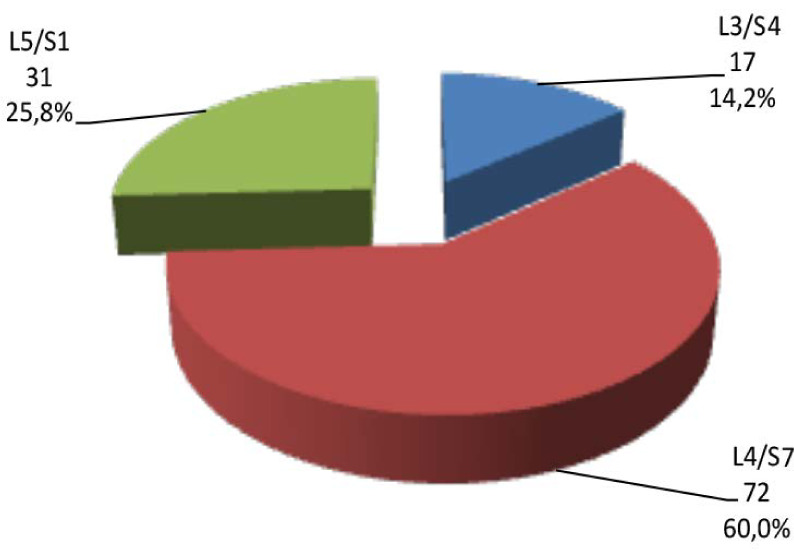
Frequency distribution of patients according to the level of disc involved *L5/S1 indicates fifth lumbar vertebra/segment 1 *L3/S4 indicates third lumbar vertebra/segment 4 *L4/S7 indicates fourth lumbar vertebra/segment 7

**Figure 2:**
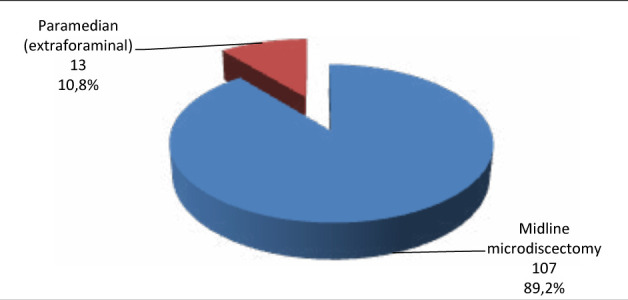
Frequency distribution of patients according to type of surgical approach

The sociodemographic characteristics of patients with lumbar disc herniation enrolled in this study are shown in Table 1. The frequency distribution of patients according to the level of disc involved is shown in Figure 1. The frequency distribution of patients according to the type of surgical approach is shown in Figure 2. The duration of disease, duration of hospitalization, and duration of post-surgical follow-up are shown in Table 2. The evaluation of nerve root compression and muscle strength is shown in Table 3. The correlation between the duration of hospitalization and the duration of post-surgical follow-up to other characteristics is shown in Table 4. There was no significant correlation to age, gender, level of education, income, level of disc involved, and type of surgical approach (p>0.05); however, there was a significant positive correlation to body mass index and duration of disease (p<0.05).

**Table 1:** Sociodemographic characteristics of patients with lumbar disc herniation enrolled in this study

Characteristics	Results
**Number of cases**	120
**Age (years)**	
Mean ±SD	47.39±10.03
Range	39-61
**Gender**	
Male, n (%)	75 (62.5%)
Female, n (%)	45 (37.5%)
**BMI (kg/m^2^)**	
Mean ±SD	29.29±8.72
Range	21-39
**Level of education**	
No formal education, n (%)	31 (25.8%)
Primary or secondary, n (%)	60 (50.0%)
Tertiary, n (%)	29 (24.2%)
**Income**	
Low, n (%)	19 (15.8%)
Intermediate, n (%)	85 (70.8%)
High, n (%)	16 (13.3%)

BMI: body mass index; SD: standard deviation; n: number of cases

**Table 2:** Duration of disease, duration of hospitalization, and duration of post-surgical follow-up

Characteristic	Result
**Disease duration (years)**	
Mean ±SD	3.09±2.01
Range	1.5-8
**Postoperative hospitalization period (days)**	
Mean ±SD	3.51±2.08
Range	2-7
**Outpatient follow-up period (days)**	
Mean ±SD	125.92±35.08
Range	61-189

SD: standard deviation

**Table 3:** Evaluation of nerve root compression and muscle strength

Characteristic	Number of cases	%
**Nerve root compression**		
Grade I	37	30.8
Grade II	59	49.2
Grade III	24	20.0
**Muscle strength MRC**		
Grade 4 (Mild)	62	51.7
Grade 3 (Moderate)	48	40.0
Grade 0-2 (Severe)	10	8.3

The Medical Research Council (MRC) Scale for Muscle Strength

**Table 4:** The correlation of duration of hospitalization and duration of post-surgical follow-up to other characteristics

Characteristic	Duration of hospitalization		Duration of post-surgical follow-up	
	r	p-value	r	p-value
**Age**	0.193	0.203	0.186	0.304
**Gender**	0.201	0.319	0.291	0.098
**BMI**	0.391	0.021*	0.421	0.011*
**Level of education**	0.102	0.309	0.137	0.417
**Income**	0.112	0.289	0.236	0.217
**Level of disc involved**	0.183	0.213	0.206	0.314
**Type of surgical approach**	0.211	0.329	0.281	0.198
**Duration of disease**	0.408	0.013*	0.371	0.024*

* : significant at p≤0.05

## DISCUSSION

Lumbar disc herniation is a common spinal disease that frequently affects young and middle-aged patients [16]. Therefore, it is important to provide patients with appropriate information about the disease, including treatment options, prognosis, the side effects of treatment, and especially the length of treatment.

In this study, we explored the correlation of duration of hospital stay and duration of follow-up of disease to several sociodemographic and operative characteristics in patients undergoing surgical intervention to address lumbar disc herniation. The results revealed no significant correlation to age, gender, level of education, income, level of disc involved, and type of surgical approach (p>0.05); however, there were significant positive correlations to body mass index and duration of disease (p<0.05). The body mass index plays a significant role in the duration of hospitalization and duration of follow-up. This implies that weight reduction should be considered before surgical intervention. Moreover, disease duration appears to be an important factor in post-surgical intervention and duration of hospital stay, thus, early detection of lumber disc herniation and early medical and surgical intervention may reduce the need for prolonged post-surgical follow-up.

Age and gender were not significantly associated with disease follow-up duration. This contrasts previous research revealing that men and women respond to lumbar disc herniation (LDH) surgery differently [17-19]. Age-related gender variations in outcomes have also been noted [20]. Male patients most typically have greater muscle mass and strength than female patients, which may have an impact on the outcomes. Rahme *et al*. [17] revealed that the female gender and physical inactivity are correlated to unfavorable prognostics for LDH surgery. Female patients typically exercise less than male patients. Rehabilitation has been shown in several studies to enhance surgical outcomes [21].

No significant association was observed between the level of education or income and hospital stay or follow-up duration, and no previous reports about such an association were found.

## CONCLUSION

In conclusion, the body mass index and duration of disease are significant predictors of prolonged postoperative follow-up and hospital stay duration. In contrast, age and gender were not significantly associated with disease follow-up duration.
